# Private and public values of innovation: A patent analysis of synthetic biology

**DOI:** 10.1016/j.respol.2019.103875

**Published:** 2020-02

**Authors:** Barbara Ribeiro, Philip Shapira

**Affiliations:** a Manchester Institute of Innovation Research, Alliance Manchester Business School, University of Manchester, United Kingdom; b Manchester Synthetic Biology Research Centre for Fine and Speciality Chemicals (SYNBIOCHEM), University of Manchester, United Kingdom; c School of Public Policy, Georgia Institute of Technology, United States

**Keywords:** Value mapping, Patent documents, Patenting, Synthetic biology, Private value, Public value

## Abstract

Emerging science and technology fields are increasingly expected to provide solutions to societal grand challenges. The promise of such solutions frequently underwrites claims for the public funding of research. In parallel, universities, public research organizations and, in particular, private enterprises draw on such research to actively secure property rights over potential applications through patenting. Patents represent a claim to garner financial returns from the novel outcomes of science and technology. This is justified by the potential social value promised by patents as they encourage information sharing, further R&D investment, and the useful application of new knowledge. Indeed, the value of patents has generated longstanding academic interest in innovation studies with many scholars investigating its determinants based on econometric models. Yet, this research has largely focused on evaluating factors that influence the market value of patents and the gains from exclusivity rights granted to inventions, which reflect the private value of a patent. However, the patent system is a socially shaped enterprise where private and public concerns intersect. Despite the notion of the social utility of inventions as a patenting condition, and evidence of disconnection between societal needs and the goals of private actors, less attention has been paid to other interpretations of patent value. This paper investigates the various articulations of value delineated by patents in an emerging science and technology domain. As a pilot study, we analyse patents in synthetic biology, contributing a new analytical framework and classification of private and public values at the intersections of science, economy, and society. After considering the legal, business, social and political dimensions of patenting, we undertake a qualitative and systematic examination of patent content in synthetic biology. Our analysis probes the private and public value propositions that are framed in these patents in terms of the potential private and public benefits of research and innovation. Based on this framework, we shed light on questions of what values are being nurtured in inventions in synthetic biology and discuss how attention to public as well as private values opens up promising avenues of research in science, technology and innovation policy.

## Research highlights

• Patent documents are a signalling mechanism about innovation values.
• Extant patent valuation literature tends to overlook the public value of innovation.
• Both private and public value propositions are found in patent documents.
• Public value propositions are less frequent but more diverse.
• Analysing private and public values in patents offers innovation policy insights.

## 1 Introduction

Research and innovation are increasingly expected to provide solutions to societal grand challenges (Stilgoe et al., 2013; Ribeiro et al., 2017). Yet, scepticism is raised about the benefits and social accountability of science and technology (Hessels and van Lente, 2008; Tyfield, 2012) and emerging technologies are reproached for not delivering on promises (Hopkins et al., 2007; Gittelman, 2016). Nonetheless, research and innovation performers increasingly use notions of societal needs, challenges, and public benefit as value claims to justify public sponsorship (Youtie and Shapira, 2017). At the same time, universities, public research organizations, and – most notably – the private sector actively and increasingly seek property rights over potential applications through patenting, with consistent worldwide growth in patent filings in recent decades (WIPO, 2018).

As a form of protection of intellectual property, governments rationalize patent systems as a mechanism to correct market failure and to incentivise investment in R&D. Such a mechanism is based on the presumption that unprotected free knowledge will deliver sub-par or even null financial returns to its creators, which in turn would lead to under-investment in research and innovation, under-productive markets, and poorer economic and social outcomes. A patent is a form of protection of invention that works by ways of excluding actors other than patent holders from reproducing or using the invention, unless its license is shared, sold or expires. Along with additional mechanisms such as prizes and research contracts, patents aim at incentivising R&D (Wright, 1983) and are one of various means through which inventors can appropriate returns from new products and processes, with a variable role among different technologies (Nelson, 2012). As a policy instrument, patents may also serve other objectives, such as signalling innovation to attract investments and promoting markets for technology (Encaoua et al., 2006).

The “value” of patents has been a longstanding subject of academic interest for economics of innovation, business and management and policy studies, as patents represent a claim to garner financial returns from the novel outcomes of science and technology. A number of scholars have investigated the potential determinants of patent value based on econometric models (e.g. Sellers-Rubio et al., 2007; Bessen, 2008, 2009; Ernst et al., 2010; Suzuki, 2011). Quantitative approaches in the fields of economics and bibliometrics have been deployed to address the complex task of measuring the commercial value of patents (for recent refinements to these kinds of approaches, see Fischer and Leidinger, 2014).

This burgeoning body of research has largely focused on evaluating the various factors that may influence the market value of patents, that is, the financial returns from protected inventions. Understood as economic gains from exclusivity rights granted to such inventions, these reflect the *private value* of patents (Gronqvist, 2009). Yet, patents also embody claims related to the *public value* of inventions by encouraging information sharing, further R&D investment, and the useful application of new knowledge (Machlup, 1958). The patent system is a socially-shaped institution where private and public concerns intersect (Gittelman, 2008; Sunder Rajan, 2012). However, despite the policy importance of considering the public value of science, technology and innovation (McNie et al., 2016) and the relationship between the social utility of inventions and patenting (Calvert, 2004; Radder, 2004; Mossoff, 2007), less attention has been paid to other interpretations of patent value than the financial value of patents to inventors and owners (Calvert, 2004).

In this paper, we investigate value claims (i.e. propositions) embedded in patent documents to explore the articulation of a series of “private” and “public” values of inventions. Taking synthetic biology as an exemplar of a field of research and innovation motivated by societal challenges, we explore the meaning of value propositions at the nexus of economic interests, public and stakeholder interests, and strategic behaviour to address questions of interest to science, technology and innovation policy. Synthetic biology is an emerging domain that is justified by expectations that it will contribute to a range of societal needs including environmental protection, reduced or higher value use of non-renewable natural resources, enhanced human welfare, and economic development (Shapira et al., 2017; Ribeiro and Shapira, 2019). The empirical part of the paper is based on a content analysis of US patent documents in this field and focuses on unpacking private and public value propositions (as articulated and embedded in these documents) of synthetic biology inventions.

With governments, businesses and academics increasingly relying on broad patent mapping and counting exercises to examine the innovation landscape (Trippe, 2015), detailed analyses of private and public values of inventions should not be left aside. While acknowledging the well-established arguments about both the limitations and utility of using patents to assess innovation (Pakes and Griliches, 1980; Pavitt, 1985), we argue that by shedding light on the kinds of values that are being nurtured in inventions, we respond both theoretically and empirically to earlier calls for a deeper qualitative understanding of innovation ecosystems (Nelson 2012). We also respond to recent calls for scholars to contribute with critical thinking on public value beyond the market failure paradigm, elucidating how private and public actors ‘innovate to solve societal problems’ and how we might ‘nurture and evaluate public value’ (Mazzucato and Ryan-Collins 2019).

This paper is organised as follows. The next section situates the term “value” semantically and conceptually, both in relation to the field of patent valuation, where it has been historically mobilised the most, but also extending it to other conceptualisations of value outside patent valuation. Section 3 sets out the framework that informed the patent analysis, introducing our operational definition of private and public values in patents in the context of the patent system and patenting processes. Section 4 describes the content analysis of patents’ full-text in the field of synthetic biology and presents the results of the application of our analytical framework. Finally, Section 5 discusses the findings, highlighting the contribution of the study to advancing the theory of private and public values of innovation, the methods for mapping those values in the field of patents and their implications for science, technology and innovation policy research.

## 2 Theoretical background

In ethics and political philosophy, values have been defined as a set of beliefs and principles that influence or guide people's' actions. The concept has been historically conceptualised as the universal or fundamental moral tenets which should mediate one's own pursuit of happiness (Kant, 1974); in Rawlsian terms, values refer to the basic principles of a socially just and fair society built on mutual respect and reciprocity (Brooks and Nussbaum, 2015). In this context, the idea of envisaging a set of universal values to govern moral conduct has been extensively debated as well as the challenges of negotiating shared (or fundamental) values amongst different individuals, organisations and societies (Bok, 1993).

Analyses of values as fundamental principles or the ethos followed by individuals and organisations have been very prominent in fields such as bioethics (Childress, 2017). Studies on people's' values that go beyond their application as “moral lens” extend, however, to a range of other disciplines interested in their relationship with broader social dynamics, including public management (Moore, 1995; Hartley et al., 2017) and the management of innovation (Sai-Manohar and Pandit, 2014). Here, a more general definition of value as the “worth” (or the value, as a noun) of something in terms of its subjective importance to different actors is proposed. In other words, something becomes valuable as people value it. To value, as a verb, thus indicates the action of assessing the worth of something. Importantly, this worth may or may not correspond to its financial value. For example, looking at academic and industrial scientific practices, Haeussler (2011) deploys the Bourdieusian notion of social capital to explain how the decision on sharing scientific information is shaped not only by a notion of “competitive value” but also by perceptions of reciprocity, recognition, and trust amongst peers. The value of information sharing is therefore a product of entangled dimensions of social capital. As well as having an impact on information sharing, these dimensions also “moderate the effect of competitive interest considerations on a scientist's willingness to share information”, and this happens in both industrial and academic settings (Hauessler, 2011: 116).

More commonly, however, value is conceptualised in terms of the financial return, monetary worth or market price of something. In the context of value appropriation and creation by firms, for example, Jacobides et al. (2006) show how “industry architectures” in terms of economic behaviour influence the appropriation of value from innovation and identify strategies for creating value from innovation by “asset appreciation”. For the authors, both objectives relate “to the question of profiting from innovation” (Ibid, p. 1201) and entail the idea that the benefit of innovation – in this case, to the firm innovating – is best measured as economic advantage in terms of financial return.

In the context of patents, i.e. a form of protection of inventions (and innovation), value has been conceptualised in different ways in the literature. The following sections (2.1 and 2.2) introduce two possible conceptualisations of a patent value; first, in terms of the most prevalent understanding of value assessed in terms of financial returns to patent holders, i.e. private value; and, second, as a marginal, but emergent notion of creation of value beyond financial returns which is directly extended to society, i.e. public value.

### 2.1 The private value of patents

Innovation scholars have had a longstanding interest in measuring patents’ value in terms of their financial return to patent holders, as well as understanding the multiple factors that may influence the value of patent-protected inventions (e.g. Guellec and van Pottelsberghe de la Potterie, 2000; Sellers-Rubio et al., 2007; Gittelman, 2008; van Zeebroeck and van Pottelsberghe de la Potterie, 2011). For example, exploring the case of software patenting, Hall and MacGarvie (2010) define the private value of a patent as the financial benefits obtained by patent holders from introducing an invention in the market. Besides being intrinsically valuable, they also suggest that patents can be an indicator of the private value of innovative outputs.

In the patent valuation literature, the probability that a patent will be economically successful and bring in economic benefits to patent holders has been associated with their novelty, technical features, impact on inventive activity, and strategic competitive utility. Indicators used in economic assessments of patent value include, among others, patent scope, family size, the number of backward and forward citations, the number of claims, and renewal information (OECD, 2015, Chapter 2). These indicators are used to assess latent aspects that are believed to influence a patents’ private value. For example, in a study of patent value determinants in the semiconductor industry, Reitzig (2003) found that the main factors influencing a patent's ‘economic value’ to a firm are its capacity to deliver knowledge of technical importance, its position in a portfolio, its learning value and the difficulty to invent around. In a later study, Reitzig (2004) suggests that, despite numerous efforts toward the operationalisation of a patent's value, further understanding on the complex relationship between indicators, determinants and the commercial success of patents is needed. The author therefore proposes other indicators besides the technical ones mentioned above, designing variables included in the text of patent documents, such as the number of words for describing the state of the art and the technical problem or the number of mentioned technical advantages of a given invention. For Reitzig (2004), the quantification of these claims could be used as a metric for assessing a patent's value, which in turn can be determined through “(observable) effects on prices, costs and sold quantities of patent-protected products by the owner” and/or “(unobservable or counterfactual) effects on the proprietor's competitors” (Ibid., p. 940).

Most patent valuation studies have taken a micro-scale, technical approach to the assessment of patents’ private value. That is, they have advanced the methods for quantifying patent value based on a series of indicators embedded in patent data. Although using patent data to measure patent value has proven useful, in the context of new patenting strategies, inflation in patent application sizes, and increase in the size of patent databases, the approach does not come without challenges (Van Zeebroeck, 2011). This has motivated alternative models and conceptualisations for assessing patents’ private value. These have included, for example, the evaluation of the potential of “a market for the patented invention” (Ibid, p. 34), and the connection between patents’ value to notions of technological and business value, introducing novel dimensions such as linkages between inventions and academic activities and even inventors’ motivations (Suzuki, 2011).

### 2.2 The public value of patents

Looking at the creation and measurement of private value from marketable knowledge produced by firms, around four decades ago Pakes and Schankerman (1979) also alluded to the notion of social value (or social return) as an opposed, but complementary, perspective to that of the private return on investment. They built on Hirshleifer (1971), for whom there was a “crucial contrast” between the notions of private and social forms of knowledge and information, the former applying to the benefits generated to a single individual; the latter extending to a larger community of individuals (Ibid., p. 564). The “public value” (used here as a synonym for social value) of patents is a much less articulated notion in the patent value literature (Suzuki, 2011), and one often proxied, ironically, by private value. In early developments of the patent valuation literature, for example, Trajtenberg (1990) conflated “social value” with the “economic success” of patents. For the author, the importance of a patent for an innovating firm is linked to its “economic significance”. Looking at medical innovation in the field of computed tomography, Trajtenberg conceptualizes the social gains of innovation not in terms of the benefits to patients from improving diagnostic methods, but essentially as the “bulk of gains” to firms based on sales data (Ibid, p. 178). Similar assumptions are built in recent studies, where a patent's social value has been equated to “expected profits and expected consumer surplus from the patent (…) litigation costs and precautionary (infringement avoidance) costs” (Hylton and Zhang, 2017, p. 45). Of importance here is the fact that, although these studies do engage with the notions of social value and consumer surplus – as analogous terms to the benefits translated from inventions to society – they do not disentangle these notions. The meaning of consumer surplus is not investigated nor challenged by these studies. Moreover, in a perfectly functioning market the private value of something would equal its public value, but we know that this is not the case in reality.

In this context, Bozeman (2002, p. 146) observes that “what most economic approaches to public value have in common is that they are less a reflection of public value than of the private value of public things.” Resonating with this argument, these articulations of patents’ public value suggest, even if only indirectly, that the generation of economic benefits to patent holders is a type or function of social value and that broader public benefits are automatically translated from an ever-growing marketplace. Baron and Delcamp (2012), for example, define the social value of a patent as “the contribution of the underlying invention to social welfare, including both future technological developments and the value of current commercial applications” (Ibid, p. 582). Indeed, the authors use the same indicators used in the assessment of private value (e.g. forward and backward citations, number of claims, family size etc.) to that of patents’ public value. The connection to technological development echoes with the expectations of value generation to society from disclosure or, in some cases, the possibility of using certain components of a patented invention (Cohen and Lemley, 2001). Nonetheless, once there are challenges to the assumption that private market economies equate to social welfare (Meckstroth, 2000; Freeman, 2010), questions also arise about some of these interpretations and definitions of patents’ social value present in the patent valuation literature. In the next part of the paper, we pursue other ways of interpreting the public value of patents.

#### 2.2.1 Reframing the public value of patents

The public dimension of patents has been an object of scrutiny for a long time, arguably since governments around the world started to implement and regulate their patent systems. As early as 1813, Thomas Jefferson, the first patent examiner of the US patent system, voiced concerns over the power of monopolies and how they could be detrimental to society, producing “more embarrassment than advantage” (Jasanoff, 2016: 183). In countries such as Germany and France, compulsory public interest licensing laws were passed in the early decades of the 20th century. This was fuelled by concerns over the accessibility to basic goods enjoying private protection, such as technologies and pharmaceuticals needed in wartime, which motivated public interest clauses in patent law (Parthasarathy. 2017). In more recent developments, the compulsory licensing of patents of pharmaceuticals for export to low-income countries with public health challenges, for example, was included in European law in 2006 under the Agreement on Trade-Related Aspects of Intellectual Property Rights (TRIPS agreement) that followed from the Doha declaration of 2001 (EC, 2006). Indeed, patents have been a longstanding subject of controversy (Machlup and Penrose, 1950), one that has been largely framed around a conflict between moral values and private intellectual property. This branch of research has enjoyed great attention especially from academics interested in examining the medical and therapeutic sector and processes of commodification of life (see Jasanoff, 2016).

Since the first patent systems were implemented, articulations of social responsibility in the governance of patents have been largely conceptualised in terms of the role of states in preventing public harm and avoiding hurting basic moral values. As it reads in the section on *ordre public* (or public policy) and morality of the European Patent Office (EPO) guidelines for patent applications examinations (EPO 2016: 75):

The application must not contain statements or other matter contrary to “ordre public” or morality. Such matter may be omitted when the application is published, the published application indicating the place and number of words or drawings omitted. (…) this will entail a cursory examination to ensure that the application does not contain the following prohibited matter: statements constituting an incitement to riot or to acts contrary to “ordre public”, racial, religious or similar discriminatory propaganda, or criminal acts and grossly obscene matter.

This regulating role of states in patents governing has been mostly a reactive one. Although debates have been theorised on broad ethical grounds (e.g. Schuklenk and Ashcroft, 2002; Williams-Jones and Graham, 2003; Forsberg et al., 2017), they have been focused on preventing public harm and avoiding value conflicts. This has outweighed critical analyses of the role of innovation in promoting public benefits, their alignment to societal needs and the distribution of such benefits in the case of patents. Such a broadened scope of analysis is the basis of our definition of the public value of patents, namely: the *intended* contribution of patents to society as an extended measure of the value of inventions that goes beyond their private value to inventors and that does not take at face value the generation of benefits to society from incentivising innovation and markets alone.

The idea of value as a measure of social utility or usefulness of a certain activity, product, or a process for human society, has deep roots in political economy. For instance, Marx's conceptualisation of social value emerged as a critique of the value paradigm of extracting surplus value for the accumulation of capital by the capitalist bourgeoisie (Amin, 2013). It is an idea also exemplified in the case of medical ethics. Jones (2016) documents a late 19th century tension that arose between practitioners who defended the primary role of an ethical medicine as one in “service of humanity” and those who would primarily see it as a commercial endeavour and source of individual profits (Ibid: 603). Machlup and Penrose (1950), in discussing the history of patent controversy, allude to this notion, while explaining one of the main arguments behind patent systems, that of the need for inventors to render “services [that] are useful to society”, so that “society secure to him [the] reward for his services in proportion as these services are useful to society” (Ibid: 10). The idea is further noted by Jasanoff (2016) who writes, with regards to the principle that emerged around patenting in the early modern period, that “those who invent something of value to the state *(and, later, of public value)*” were considered to deserve exclusive rights to their inventions (Ibid: 183, emphasis from authors).

Public value has been notably theorised in research and science policy evaluation, especially by Bozeman. Although considered as correspondent concepts, Bozeman (2007) distinguishes public interest from public values. He refers to the former as the outcomes that best serve the well-being of society and the latter as a set of values that provide normative consensus about the rights and benefits to which citizens are entitled, the obligations of citizens to society and the principles that should guide governments policies (Ibid: 17). Conceptually, in their operationalization of a framework called public value mapping, Bozeman and Sarewitz (2011) approach the question of the public value of science from the perspective of impacts on society. For the authors, this is an *ex-post* appreciation of the capacity of research (or science outcomes) to produce social change, something that goes beyond mainstream evaluations of scientific and economic impact. Here, they criticise how advances in social goals and quality of life have been included in the economic value cluster of science policy. This resonates with the problematic assumption discussed earlier, that economic growth derived from the advancement of science, technology and innovation automatically translates into shared public benefits and an increase in well-being (see Woodhouse and Sarewitz, 2007). Bozeman and Sarewitz (2011) examine the public values embedded in US science policy. They find that “the breadth of values expressed (…) is significantly wider than the breadth of values directly pursued or assessed”, with public values being “often subverted, reinterpreted, and subjugated to the science-economy axis” (Ibid: 4). Their diagnosis is one of misalignment between the claims and promises made by science, and those who regulate and fund it, with the actual outcomes of this science. Analogously, one can apply this reasoning to the case of patents and ask what are the public values embedded in research and innovation reflected in patents, many times in terms of imagined societal needs, and how they might align with the social impacts of these inventions.

In the context of patenting, Calvert (2004) engages with the concept of utility to offer a critical examination of such a requirement in patenting processes. She highlights the importance of the social context of inventions in the field of genomics and that of asking questions about the meaning of utility and the distribution of benefits. While Calvert (2004) does not adopt the private and public value terminology, she starts a much-needed discussion on what the broader social utility of inventions might mean and what kinds of values (referred by the author as “types of utility”) are being embedded in gene patenting. Adding to these rare accounts of public value in patent analyses, Chojnacki and White (2013) explore social utility arguments in genomics patents. Examining the famous case of a controversy over patenting breast cancer genes, the authors note the potential uneven distribution of benefits between breast cancer patients – the assumed primary beneficiaries – and patent-holders. Here, the public value of an invention is associated with its social utility extended to a range of actors (and nations) with different, sometimes competing, interests and concerns.

## 3 Exploring private and public values in synthetic biology patent documents

The conceptualisations of value discussed in the previous section deal mostly with the singular form of the term, i.e. as a synonym for the worth of something (be that worth of a private or public nature). This worth is dependent upon the different *qualities* of an invention or, in other words, the types of utilities it embeds. These qualities may benefit the owners of a patented invention and its users, or civil society, or both. In this section of the paper, we explore these qualities as they are articulated in the full-text of a patent document (i.e. applications and granted patents). Building on the conceptualisations of value discussed earlier, we classify and differentiate them in terms of private and public value propositions. Through a qualitative and systematic content analysis of a set of patent documents, we illustrate and introduce a new way of conceptualising and analysing the values of innovation. As we will show, the private and public values of an invention are represented by statements about the qualities of that invention (i.e. value propositions). These claims can be found in the full-text of patent documents in the form of statements about the potential industrial, scientific, environmental and social benefits, among others, of an invention. For this part of the study, we use a set of patent documents from synthetic biology. We provide a brief introduction to the field of synthetic biology, justifying why it can be a useful domain of study in the context of this discussion, and then present the methodology that guides the patent analysis.

### 3.1 Domain of the study

Synthetic biology is an emerging interdisciplinary field of biotechnology. Through a combination of approaches from biology, chemistry, computer sciences and engineering (Shapira et al., 2017), synthetic biology seeks to synthesise “complex biological-based or biologically-inspired systems to display functions that either mimic nature or go beyond nature” (RSC, 2008). The narrative underpinning synthetic biology is one that is particularly fuelled by promises of solutions to address societal challenges in areas ranging from human health to environmental sustainability (Hellsten and Nerlich, 2011). The field is thus characterised by a focus on translational, application-oriented research, with a view to bringing scientific advancements to the market (Eils et al., 2015). Target sectors for synthetic biology include, for example, pharmaceuticals (e.g. anti-malaria and anti-cancer drugs), food (e.g. flavouring compounds) and energy (e.g. biofuels), among others (Clarke and Kitney, 2016; Tyagi et al., 2016; Wurtzel and Kutchan, 2016).

As argued by Bensaude (2013), the high expectations around synthetic biology are not only part of its rhetorical apparatus, but a constituent of its own methods and the values followed by the scientific community attached to it. In this context, the field has attracted the attention of scholars interested in debating and shaping its orientation towards the future (Frow and Calvert, 2013). Given that synthetic biology entails a bundle of novel technologies, which in turn bring with them a great deal of uncertainty on their ethical, legal and social aspects, it is a also a controversial field. This has motivated research in the areas of responsible research and innovation (RRI) and technology assessment applied to synthetic biology, which has highlighted approaches to evaluate and shape the development of applications in the field (Wiek et al., 2012; Macnaghten et al., 2016). For example, Lentzos (2009) indicates a series of emerging concerns, including questions around fairness, equality and progress. Importantly, the author finds that “synthetic biology would not have generated the investment of time, energy and resources that it has if it was not seen to have a social utility, and if that social utility was not in some ways seen to have a public value and a commercial value” (Ibid, p. 311).

Synthetic biology thus offers a prime example of an area of science, technology and innovation where promises intersect with private and public value. As mentioned above, it is also one that emphasises its translational focus, from R&D to market. In this context, it is worth examining more closely what kinds of value propositions that anticipated applications of synthetic biology embed in terms of their social utility for both private and public interests.

Patent documents, as sites in which novel applications of synthetic biology are described in detail, offer an interesting and relevant setting for exploring private and public values associated with the field, both in terms of the specific invention being patented and the field in general. Although the ethical and legal aspects of patenting in synthetic biology have been addressed by some scholars (Rai and Boyle 2007; Calvert 2008; 2012), few analyses of synthetic biology patents are available in the literature. Those that are available have focused on describing the patenting landscape in terms of its general trends or in relation to specific sub-fields such as chemical production (Oldham et al., 2012; van Doren et al., 2013; Carbonell et al., 2016; Oldham and Hall, 2018; Shapira and Kwon, 2018). To the best of the authors’ knowledge, the analysis presented below is the first to consider the elaboration of private and public value propositions for patents in synthetic biology as well as other technological domains through a full-text content analysis of patent documents. This is an exploratory pilot study that is useful for designing and testing the operationalisation and measurement of concepts of patent value.

## 4 Research design and method

### 4.1 Data collection

A total of 102 patent documents published by the United States Patent and Trademark Office (USPTO) in the field of synthetic biology between 2011 and 2014 (inclusive) were retrieved from the Lens database (https://www.lens.org). These were identified within a comprehensive sample of patent documents that reflected a global search strategy for synthetic biology patents (Kwon et al., 2016). After checking for relevance, ninety-seven (*n* = 97) patent documents were selected for full text analysis and manual coding (five documents were excluded as they were not directly related to synthetic biology developments). The amount of data in the documents analysed in our study was considerable: for the 97 patent documents, the full patent text (not just the metadata) totalled about 8700 pages – an average of 90 pages or 33,600 words per document. The size of the sample and the amount of text data is appropriate for the purposes of an exploratory study focused on a qualitative analysis of the content of the full-text of patent documents. Our patent sample size is comparable to those observed in case studies used in earlier patent research (Mowery and Ziedonis, 2002; Reitzig, 2003; Sternitzke, 2013).

The selected documents included both granted patents and patent applications. It should be noted here that the content of the full-text does not vary between documents belonging to the same simple patent family, which covers the same invention, as opposed to extended patent families, which do not necessarily cover the same invention as documents may lack a common priority application (Martínez, 2010; Trippe, 2015). To avoid duplication of the content of the full-text, each of the documents selected in this study is representative of a different simple patent family. The analysis excluded the claims section of the selected documents given that there is substantive content variability in this section among documents belonging to the same invention. Our corpus therefore represents a purposive collection of documents for a patent analysis that prioritises the representativeness of different inventions.

### 4.2 Data analysis

We applied content analysis methods (Bryman, 2012) to the text of each document in our patent set to explore embedded private and public value propositions and the problem framings to which these propositions are related. A patent document contains a series of standardized sections including data to identify and classify the document according to the specific patent office and international classifications, background information on the invention, and detailed descriptions of the features of the invention. The document also contains claims (included in the specific claims section) which aim to determine the breadth or scope of the invention and set the boundaries of the patent protection, and may include drawings. Patent documents have standardised structures, although there is variation in terms of the length of the different sections and their specific content. For US patents, there are requirements in terms of the form of the claims section which defines the scope of legal protection sought for a specific patent. There is less guidance for the writing of other sections, although in general “adequate disclosure” is required to “[ensure] that the public receives something in return for the exclusionary rights that are granted to the inventor by a patent.” (USPTO, 2018 Section 608).

Our approach to content analysis of the patent text was qualitative (Macnamara, 2005) with deductive and inductive approaches to coding (Sandelowski, 2000; Hsieh and Shannon, 2005; Gioia et al., 2012). We used QSR's NVivo 11 as support software for an analysis performed by two different coders at two different stages. In the first stage, the first author coded the sample until coding saturation was reached and a protocol was produced. Following general inter-coder reliability practice in content analysis (Campbell et al., 2013; Lovejoy et al., 2014), a second coder then tested the protocol in 10% of the sample, a sub-set comprised of random patent documents representative of different years. Such an approach for multiple coding has been adopted elsewhere to support the subsequent calculation of intercoder reliability in content analysis (Sillanpää and Laamanen, 2009; Verstegen et al., 2018). Intercoder reliability was calculated, returning an average Kappa coefficient of 0.83 (with a 95% confidence level range of ±0.05). Guidelines for interpreting Kappa indicate that this is a substantial to almost perfect (Landis and Koch, 1977, p. 165) or excellent (Fleiss et al., 2003, p. 604) level of agreement, beyond chance. Given that agreement between coders was high, the protocol was used by the first coder for coding the remainder of the sample. A final check of the full coded content was performed using the protocol for refining the categories.

The content analysis focused on private and public value propositions in relation to synthetic biology inventions and their associated problem framings. Sentences and paragraphs found in the full-text of patent documents were used as units in the analysis of manifest content of communication, i.e. information that is explicitly conveyed by the text (Gray et al., 2007). The deductive part of the analysis drew on the authors’ definitions of the categories of private value propositions, public value propositions and problem framings (see below), where coded text from patent documents was grouped within each of the categories. Sub-categories linked to the initial categories (see sub-sections of Section 5) emerged from the inductive part of the analysis, which focused on coding the content of the initial categories. Value propositions are understood as written articulations of the embedded values of inventions. Building on the Bozeman and Sarewitz (2011) empirical strategy for the analysis of values, we assumed that private and public values can be identified in “articulations of desirable states toward which progress can be assessed” (Ibid: 13). In patent documents, we name these articulations value propositions given that they reflect desired or intended, but not necessarily realisable, goals of a given invention. Propositions related to private value suggest the commercial and industrial qualities of an invention or, in other words, they correspond to features that aim to make an invention attractive to markets. Conversely, those related to public values are illustrated by statements regarding the potential social benefits of an invention, beyond their value to patent holders. The different problem framings put forward as a background to these values were also analysed. The articulation of problems to which an invention responds provides information on the context and motivations behind the invention and is commonly found in the background section of a patent document. There is a diversity of problem framings among inventions and the same invention may mobilise multiple problem framings.

## 5 Results

The majority of the patents in our sample were assigned (by USTPO using International Patent Classes) into the biochemistry, microbiology, enzymology, mutation and genetic engineering classification. Other prominent classes included petroleum and gas industries, organic chemistry and agriculture. These groupings reflect the diversity of the field of synthetic biology in terms of the multiple disciplines on which it draws its methods and the main areas of interest of its target industries. Documents published in the years of 2011 and 2012 comprised 58% of the sample, while 42% of documents were published in 2013 and 2014. Ten of a total of 54 organisations indicated as patent assignees formed half of the sample (owning 52 patents). Amongst patent owners, nine (or 17%) represented public universities and organisations. Fifty patent documents (or 52%) corresponded to inventions that had received partial of full public funding support for their development. The sections below describe the results for each of the categories that emerged from the content analysis. The relationship between the categories is assessed through the number of sources (i.e. patent documents) they share. In other words, while the coded text will be different for each category, the same document may include multiple categories.

Apart from problem framings, the results of private and public value propositions are presented in Tables 1 and 2. These tables summarise the categories identified (i.e. value propositions) from the content analysis. They provide definitions for each of the categories, examples of excerpts from the patent documents which illustrate each category, as well as indicate the distribution of the categories across the sample. The reader should note that this distribution corresponds to the number of patent documents, that is, single inventions, within which a certain value proposition has been identified – independent of the number of times such a proposition has been identified within the same document.

**Table 1:** Summary of private value propositions.

Category	Definition	Distribution (patent documents)	Examples
Market and industrial opportunities	The potential of the invention to enter existing markets	74% (*n* = 72)	“One of the primary markets for this oil is infant formula” (US08389808B2)
Cost and efficiency	Reduction of production costs associated with more efficient processes	48% (*n* = 44)	“The great potential of syngas as a feedstock resides in its ability to be efficiently and cost-effectively converted” (US20110223637A1)
Increasing compound yields	Improvements in compound productivity based on novel processes	36% (*n* = 35)	“This combination provides improved volumetric productivity for the fermentation” (US20120064590A1)
Upscaling production	Taking production to the commercial level	30% (*n* = 29)	“There exists a need for alternative methods for effectively producing commercial quantities of compounds such as adipic acid and carpolactam. The present invention satisfies this need” (US20130095540A1)

**Table 2:** Summary of public value propositions.

Category	Definition	Distribution (patent documents)	Examples
Scientific advancements	Contribution to knowledge production	46% (*n* = 45)	“The development of customizable recombinase system as enormous benefits for neuroscience as well as many fields of biological research” (US08450107B1)
Environmental sustainability	Contribution to environmental quality and preservation	30% (*n* = 29)	“The ability to manufacture 1,3-butadiene from alternative and/or renewable feedstocks would represent a major advance in the quest for more sustainable chemical production processes” (US20120021478A1)
Human health	Improvements in the quality of human health	24% (*n* = 23)	“Gene targeting may be used for treatment of disease. For example (…) to engineer corrections in genes that are defective due to various types of mutations” (US20110145940A1)
Food security	Avoiding competition with human food sources	6% (*n* = 6)	“Use of hydrolysate prepared from cellulosic biomass as a carbohydrate source for fermentation is desirable, as this is a readily renewable resource that does not compete with the food supply” (US20140178954A1)
Animal health	Improvements in the quality of animal health	4% (*n* = 4)	“The methods described herein can also be used to produce compositions effective to treat or prevent the disease (…) lung plague, a major pathogen of cattle, yaks, buffalo and zebu” (US20110053272A1)
Job creation	Potential to create jobs from the development of the sector related to the invention	1% (*n* = 1)	“The localization of national energy production will lead to a growing American economy, thus creating more jobs” (US20110124063A1)

### 5.1 Problem framings

As indicated earlier, problem framings provide information about the context and assumptions behind value propositions. They work as a type of scene setting mechanism whose function is to justify the need for an invention, describing its contribution to solving specific problems. Articulations of problem framings are usually found in the background section of a patent document (i.e. considering the version published by the USPTO), but can be identified anywhere in the full-text of a patent document. The levels with which a patent document focused on framing the problems in the context of an invention was varied, as well as the number of problems associated with an invention. A total of four different categories of problem framings emerged from the analysis of the data.

#### 5.1.1 Technoscientific bottlenecks

Technoscientific bottlenecks corresponded to the most prominent of all framings, being identified in 57% of the sample (*n* = 55). Given that patented inventions entail developments that necessarily embed some kind of technical novelty in relation to incumbent technologies, it is not surprising that this emerged as a leading category. The category included mentions to the capacity of the invention to solve specific scientific or technical bottlenecks. These would be framed in terms of an invention's contribution for responding to “a need in the art”, for example, for improving the capability of modified photosynthetic microorganisms to produce certain compounds (US20110250659A1). They would also include indications of advancing methods and techniques in synthetic biology and how inventions were able to fill gaps in current knowledge. As put by one patent application, “efforts to engineer new functional biosynthetic pathways in well-characterized micro-organisms such as *Escherichia coli* are still often hampered by issues such as imbalanced pathway flux, formation of side products and accumulation of toxic intermediates that can inhibit host cell growth” (US20130130347A1).

#### 5.1.2 Challenges of conventional production processes

The category of challenges of conventional productions processes is characterised by an articulation of the disadvantages of conventional methods for obtaining compounds or products of interest, particularly including economic and commercial aspects of these disadvantages. This type of problem framing was found in 56% of the sample (*n* = 54), making it the second most popular category, with 18 of these documents also articulating the previous category of technoscientific bottlenecks. As an example of articulation of these challenges, a granted patent in the field of oilseed plants states “unfortunately, there are several disadvantages associated with commercial production of polyunsaturated fatty acids (PUFAs) from natural sources (…) The oils obtained from these sources can require extensive purification to separate out one or more desired PUFAs or to produce an oil which is enriched in one or more PUFAs” (US08389808B2).

#### 5.1.3 Sustainability concerns

The third category, sustainability concerns, is related to the category of challenges of conventional processes, particularly in those documents which frame the problem in terms of reliance of petrochemical sources. 33% (*n* = 32) of the sample was coded under this type of problem framing, with 24 documents also associated with the previous category. Articulations of sustainability concerns included passages such as “the present invention produces isobutanol from plant derived carbon sources, avoiding the negative environmental impact associated with standard petrochemical processes” (US20110112334A1); or “there is a clear need for alternative routes to create both fuels and products currently derived from petroleum (…as these…) are the primary reason for climate change” (US20110124063A1).

#### 5.1.4 Increasing demand

The fourth category of increasing demand is more related to the category of challenges of conventional processes than to the other two categories. Emerging in 21% of the sample (*n* = 20), ‘increasing demand’ shares 16 patent documents with challenges of conventional processes; however, differently from the passages coded under the latter, it articulates the need for the invention drawing on the assumption of current and future demand for products of interest to synthetic biology. Typically, this problem framing is phrased in terms of increasing commercial or industrial demand, market needs, increase in population etc. One example is the framing of the need for a chemical known as butanol: “each year 10 to 12 billion pounds of butanol are produced by petrochemical means and the need for this commodity chemical will likely increase in the future” (US20140273129A1).

### 5.2 Private and public value propositions

Private and public value propositions are represented by statements that reflect expectations or promises of the invention to achieve economic and societal goals, creating direct benefits for those able to exploit the invention (i.e. patent owners) or broader societal actors. These propositions are related to one or more of the different problem framings presented above.

#### 5.2.1 Private value propositions

Our analysis finds that private value propositions are distributed among four different categories. These are described and illustrated in Table 1. All four of these categories refer to aspects connected to the financial return from the commercialization of inventions. The most predominant private value proposition is that of market and industrial opportunities, contained in about three-quarters of the patent documents. In this category, the potential of the invention to enter existing markets is highlighted. Also, indications are provided of the kinds of markets and industries the invention is suitable for, demonstrating their potential to create value to the patent owner. Private value propositions related to the reduction of production costs associated with more efficient processes are the second most prevalent category, indicated in nearly half of the patent documents. Improvements in compound productivity based on novel processes is a value category found in more than a third of patent documents. Finally, the potential to upscale production to commercial levels is a value category in just under a third of patent documents.

#### 5.2.2 Public value propositions

Public value propositions are less prominent, but more diverse in the sample compared to those related to private values (Table 2). We identify six public value categories in the patent documents that we analysed. Scientific advancement through contributions to knowledge production is indicated in 46% of the patent documents. Often included here are statements about the value for scientific fields and disciplines, suggesting the potential for subsequent scientific and technological benefits. For example, mentions are made of contributions to the modularity of biological information which can be used as building blocks for further scientific advances. This is a value contribution to the public domain that goes beyond the financial returns that might be garnered from a specific invention. The second most common category of public value is environmental sustainability through contributions to environmental quality and preservation, indicated in 30% of the patent documents. Included in this category are anticipations of lower greenhouse gas emissions, the avoidance of pollution and other negative environmental impacts associated with petrochemical processes, and reduced environmental impacts through biodegradability. Such value propositions again promise societal and environmental benefits to others and to the public sphere beyond direct financial and productivity benefits to the patent holder or subsequent specific users of the invention. Contributions to human health were anticipated in just under one-quarter of the patent documents, including novel biological methods for proteins, antibodies, and vaccines, drug delivery, and gene therapies that promised broad improvements in the quality of human health. The other public value categories of food security, improvements in the quality of animal health, and job creation are identified in a smaller number of documents than those for private value propositions. It is worth noting that animal health has a relationship to human health as some documents articulating the former also articulate the latter.

## 6 Discussion

The patenting process – including not only filing a patent but also the protection of a granted patent against infringement – is complex, resource intensive and may involve public patent authorities in different countries as well as enforcement procedures involving national and transnational courts (Encaoua et al., 2006). Indeed, inventors and assignees may choose not to patent an invention for a variety of reasons. These include the costs of applying for and enforcing a patent, the possibility of (or need for) maintaining the invention as a trade secret, or the availability of other disclosure strategies. An example of the latter is the strategic use of publishing to make an invention public and open to all, thus constraining any party from securing an intellectual property advantage. This strategy can be associated with publicly funded science (Li et al., 2015), although corporations may also pursue defensive publication to prevent others from obtaining a patent or to force competitors to narrow their patent claims (Barrett, 2002; Henkel and Pangerl, 2008).

For those who do choose to patent, Mazzoleni and Nelson (1998) identify four generic purposes that may be served by patents: to foster useful inventions; to attract investments to develop and commercialise inventions; to allow for public disclosure of inventions; and to facilitate orderly emerging domain exploration, through an opening broad prospect patent, of subsequent inventions. These are public purposes. Indeed, Mazzoleni and Nelson caution that patent protection that is too strong in favouring particular private interests can hamper economic and technological development. For inventors and their organisations, the perceived benefits of patenting include not only the commercialisation monopoly of a given product, but also to establish priority in R&D on early-stage inventions, invest and influence technological directions, pre-empt rivals, or build, for example, a patent portfolio (Gittelman, 2008), which are valuable strategic tools for R&D managers (Reitzig, 2003).

Patenting is therefore a social activity that is shaped by the motivations and interests of different actors within the frameworks and regimes set by patent systems. Patent documents are constructed not only by inventors but also by their attorneys and patent examiners, with the two last actors usually taking a leading role in the patenting process (Gittelman, 2008). Inventors often draft non-claim narratives, including description sections about the use of the invention, while lawyers are particularly concerned about the claims and prior art disclosures (Bryan et al., 2019). Lawyers and examiners, for example, may add citations to prior art at their own discretion without the involvement of the inventors, who may be unaware of the list of citations included in the final document (Criscuolo and Verspagen, 2008). While lawyers will direct efforts to guarantee a patent scope that is as broad as possible in the claims section, the main concerns and tasks of examiners will be those of scrutinising and checking the validity of the novelty claims held by inventors and their lawyers. Some would frame patenting as a bargaining process, where the different parties – those applying for and those granting a patent – negotiate the language and content of patent documents (Feldman, 2012). As a socially-shaped set of arrangements, the patent system embeds and operationalizes a series of values held by the actors within the system. Such values can be mobilised from the very start of the patenting process, all the way through the dissemination of inventions in society.

While the motivations of inventors and other actors involved in the process of writing a patent document is to frame the statements included in the claims section as precisely as possible so as to avoid infringement and maximise the scope of application of an invention, less is known about the drivers behind the crafting of the rest of the full-text. There has been analysis of the determinants of applicant and examiner prior art citations (Alcácer et al., 2009), extended through recent work on identifying use of prior knowledge through the analysis of citations to scientific literature contained in the full-text, beyond the front page citations (Bryan et al., 2019). Other studies that have looked at the full-text of patents have focused on different questions, for example using text-mining to identify keywords or technological novelty (Noh et al., 2015; Walter et al., 2017). Our analysis draws on the case of synthetic biology to show that a diversity of private and public value propositions is articulated within the full-text of patent documents. This suggests that value propositions can be instrumentally framed by inventors and actors within the patent system in ways that are thought to be able to maximize the private value of a given patent application, but also potentially its public value. As shown by the results of the analysis, each patent document will tell a ‘story’ about an invention that connects it to broader, real-world societal and/or business challenges. In order to give flesh to this story, patent documents will spell out the desirable qualities of an invention, including a series of explicit articulations of the values to which it responds. In contrast to the statements included in the claims section of a patent document, while these articulations cannot be ‘protected’ (e.g. one cannot have an exclusive right to contribute to environmental sustainability), they exert an important function of disclosing the rationale behind a given invention and the kinds of benefits to patent holders and society that are expected from its implementation.

Our analysis suggests that patents serve as potential signalling mechanisms about innovation problem framings and values in addition to the codified claims they put forward for intellectual property rights from specific inventions. The investigation of the full patent text in a set of synthetic biology patents surfaces a combination of problem framings and value propositions that provide context for the intended justification, scope and impact of the application of an invention. The synthetic biology patents addressed the four key problem framings of technoscientific bottlenecks, challenges of conventional production processes, sustainability concerns, and increasing demand. Private value propositions were put forward related to exploiting market and industrial opportunities, cost reduction, productivity, and scale up, while public value propositions emphasized advancing knowledge, environmental sustainability, and human and animal health. By demonstrating how inventions promise solutions to problems and challenges, patents also assert their potential for creating private and public value and manifest broader normative justifications for the granting of patent rights. This in turn mirrors, and may shape, expectations related to the consequences of inventions for societal challenges and benefits.

## 7 Conclusions

We emphasise that this is an exploratory study and, in so doing, also recognize limitations when interpreting the results. Our analysis draws on a set of US patents in a specific timeframe and for a particular domain. Although the assignees comprise a range of organizations and companies, there is a concentration in patent ownership. The limited sample reflects the time-demanding requirements of manual qualitative content analysis. Nonetheless, the insights from this exploration do suggest that there will be value in further research that addresses a larger-scale sample and deploys automated text mining and quantitative analytical approaches. We observe that a range of statements concerning private and public values are included in patent documents, although it is a further research task to understand the motivations that lead to the inclusion (or non-inclusion) of particular types of value propositions in patent documents. Additionally, it is outside the scope of the present study to evaluate the subsequent impact over time of the inventions analysed against the private and public values spelled out in their corresponding patent documents. Although we believe that patent documents and, in particular, private and public value propositions, can be considered as signalling devices, we accept the need for further research to examine how subsequent actions by the owners of patents correspond to these signals.

With these limitations kept in mind, we now consider the contributions and implications of the paper. This paper has empirically demonstrated that multiple values are mobilised in patent documents; it has provided a classification which can be used in the domain of synthetic biology and which could be potentially extended to other domains; it has contributed with a critical discussion of the theory and practice of patent valuation; and it has conceptualised private and public value propositions in research so as to provide with a framework that can support further studies in value mapping. Specifically, we have introduced an analytical framework into the study of patenting by probing three entangled spaces where values are mobilised by multiple actors: the context of patenting, the content of patent documents and the potential economic and social impacts of patents. We have sought to advance both the conceptualisation and methodology of understanding private and public values in innovation through an analysis and interpretation of patenting.

Some further implications flow from this work. First, our analysis has broader utility in the context of mapping trends in discourses about, and justifications for, innovation, which can be used in innovation studies for value mapping purposes. Such an analysis could inform, for example, new strategies in the private and public sector in terms of mapping trends in innovation discourse, target industries and applications. More or less standardized discourse of patent text, associated with strategic writing, may allow for the development of several taxonomies in different fields and the analysis of trends in patent discourse. We would particularly encourage future studies to focus on other emerging technology domains in addition to that of synthetic biology.

Second, the pilot work in this paper offers an added element to discussions of strategic patenting, i.e. the use of the patent process not only to protect a company's own intellectual property but also to block the acquisition or use of intellectual property by a competitor (Blind et al., 2009). There are suggestions that patents can be broadly framed (with wide scope in patent claims) at the early stages of a technological field's development, in part reflecting strategies to gain advantages over competitors (Munaria and Toschib, 2014). Arguably, considerations of public and private value propositions are unnoticed by the current literature on strategic patents, as this literature tends to focus specifically on patent claims. As mentioned before, the content of other sections of the full-text of patent documents are overlooked in patent analysis. How strategic patents with broad patent claims construct categories of problem framing and both private and public and private value propositions could be probed in further research. We would anticipate that such patents could exhibit a corresponding augmentation of their value propositions. Further research is needed to better understand the role of value propositions for strategic patenting.

Finally, the analytical framework put forward in this paper is a valuable tool for the anticipatory governance of emerging technologies such as synthetic biology and for RRI approaches interested in shedding light in the distributional aspects of the benefits of science, technology and innovation. Although it is here applied to patent documents, its use could also be extended to a series of other key secondary sources of data such as publications, reports and innovation strategies. A comprehensive understanding of the values being mobilised in the background of inventions – and science and technology development more generally – should help informing directionality in innovation policy.

## Declaration of Competing Interest

The authors declare that they have no known competing financial interests or personal relationships that could have appeared to influence the work reported in this paper.
